# Development and validation of a simple clinical prediction model for post-stroke seizures in critically ill patients: a multicenter retrospective cohort study

**DOI:** 10.3389/fneur.2026.1949946

**Published:** 2026-10-13

**Authors:** Yue Zhang, Xiaocui Yang, Yu Kang, Wenyao Cui

**Affiliations:** 1 Department of Neurosurgery, West China Hospital, Sichuan University, Chengdu, China; 2 West School of Nursing, Sichuan University, Chengdu, China

**Keywords:** clinical prediction rule, critical care, post-stroke seizure, prediction model, propensity score matching, SOFA score

## Abstract

**Background:**

Post-stroke seizures (PSS) are common in critically ill stroke patients, but ICU-specific risk prediction tools are lacking, and the independent impact of PSS on mortality remains controversial.

**Methods:**

This retrospective multicenter cohort included 6,586 adult stroke patients from 208 US ICUs in the Collaborative Research Database (eICU-CRD). Patients were randomly divided 7:3 into training and internal validation sets. The primary endpoint was coded in-hospital post-stroke seizures identified via diagnosis codes during the index hospitalization. Candidate predictors were screened using logistic regression and least absolute shrinkage and selection operator (LASSO) regression. Model discrimination, calibration, and clinical utility were, respectively, evaluated using the area under the receiver operating characteristic curve (AUROC), calibration slopes and calibration plots, and decision curve analysis. Propensity score matching (PSM) was used to evaluate the association between PSS and in-hospital mortality and length of stay.

**Results:**

The overall incidence of coded in-hospital PSS was 7.5%. Head-to-head comparison identified SOFA as the best-performing routine ICU severity score for coded PSS prediction, outperforming GCS, OASIS and APACHE II. Multivariate logistic regression analysis identified five independent clinical predictors for coded in-hospital PSS: younger age, elevated admission body temperature, pre-existing hypertension, lower mean corpuscular hemoglobin concentration, and lower Glasgow Coma Scale (GCS) score. The model achieved AUCs of 0.702 in the training set and 0.713 in the validation set, with stable performance across age, gender, and stroke subtype subgroups. A three-tier bedside risk stratification was derived, with observed coded PSS rates of 3.4, 6.8, and 16.2% for low-risk, intermediate-risk, and high-risk groups. After PSM, coded PSS was not associated with increased in-hospital mortality (adjusted OR = 0.84, *p* = 0.295) or prolonged hospital stay (mean difference = −0.30 days, *p* = 0.167).

**Conclusion:**

We developed and internally validated a simple prediction model for coded in-hospital PSS in critically ill stroke patients. Coded PSS itself was not independently associated with increased mortality. This model remains a research tool that has not undergone external validation and cannot yet guide real-world clinical monitoring or pharmacological intervention. These findings are applicable to clinically evident, coded convulsive seizures and may not generalize to non-convulsive seizure phenotypes.

## Introduction

Acute stroke remains a leading cause of death and permanent disability worldwide, responsible for approximately 11% of all deaths globally and a major driver of long-term neurological morbidity (1). With an aging population and improving acute stroke care, an increasing proportion of severe stroke patients require admission to intensive care units for close neurological monitoring, management of elevated intracranial pressure, mechanical ventilation, and support of failing organ systems (2, 3). Seizures are one of the most common and clinically important early neurological complications following acute stroke, occurring in 3 to 15% of patients depending on stroke subtype, severity, and population studied (4, 5). Post-stroke seizures (PSS), typically defined as seizures occurring within the first 7 days of stroke onset (6), are clinically concerning because they can precipitate secondary brain injury through increased cerebral metabolic demand, elevated intracranial pressure, excess neurotransmitter release, and disruption of the blood–brain barrier (7, 8). In addition, PSS frequently leads to initiation of long-term antiepileptic drug therapy, which carries significant risks including cognitive impairment, drug interactions, hematological abnormalities, and hepatic toxicity, particularly in older critically ill patients (9, 10).

The pathophysiology of PSS is multifactorial, involving acute excitotoxicity from glutamate release, blood–brain barrier disruption, cortical irritation from blood products, metabolic derangements, and inflammatory cascades (8, 11). Hemorrhagic strokes carry particularly high risk due to direct cortical irritation by extravasated blood, while large cortical ischemic strokes are also associated with elevated seizure risk (12). It is biologically plausible that general ICU illness severity scores, which quantify systemic rather than purely neurological dysfunction, may also predict PSS. Acute stroke triggers a systemic inflammatory response characterized by cytokine release (IL-1β, TNF-α), oxidative stress, and endothelial activation that can disrupt the blood–brain barrier, amplifying cortical irritability beyond the primary structural lesion (8). Multi-organ failure, as captured by SOFA (13), may further lower seizure threshold through several convergent mechanisms: hypoxemia and hypercapnia impair neuronal GABAergic inhibition and promote excitotoxic glutamate release; acute kidney failure and electrolyte disturbances directly destabilize neuronal membrane potentials; coagulopathy increases the risk of hemorrhagic transformation and cortical blood deposition; and hemodynamic instability causes secondary cerebral hypoperfusion and ischemic injury in the already vulnerable penumbra. These systemic physiological derangements act as a “second hit” that compounds the pro-epileptogenic effects of structural cortical injury (14, 15). By integrating dysfunction across multiple organ systems, scores such as SOFA may capture this systemic neuroinflammatory burden more completely than neurological-only assessments, which is why we hypothesized that multi-organ severity scores might outperform purely neurological scores for PSS prediction.

Despite the clinical significance of PSS, several critical gaps remain in our understanding of this complication, particularly in the ICU population where seizure risk is highest and clinical decision-making carries the greatest stakes. First, while several clinical prediction rules for PSS have been developed, the vast majority, including the SeLECT score, the IsCHEMiA imaging score, and recently published machine learning models, were derived and validated in general ward-based stroke cohorts with relatively low illness severity, and few have been specifically developed or validated in critically ill patients (16, 17). Critically ill stroke patients differ fundamentally from ward-based cohorts. They exhibit higher rates of organ dysfunction, receive more sedative and vasoactive medications that may alter seizure expression, and have substantially higher baseline seizure risk and mortality. Validated risk scores specifically tailored to the ICU stroke population remain scarce, and existing general-cohort tools may not perform optimally when transported to the critical care setting. Existing models also frequently rely on specialized variables such as imaging findings or EEG results that may not be universally available early in the ICU course, limiting their clinical utility for rapid risk stratification (16). Second, although multiple severity scores are calculated routinely for every ICU patient at admission, including APACHE II, SOFA, GCS, and OASIS, their relative performance for predicting neurological complications such as PSS has not been systematically compared in a large multicenter cohort (18, 19). We selected APACHE II, SOFA, GCS, and OASIS for comparison because these four scores are universally computed at ICU admission in the eICU-CRD with no additional data collection required, and they span complementary physiological domains. This selection contrasts a purely neurological score (GCS) against multi-organ dysfunction scores, allowing us to test whether systemic illness burden adds predictive value beyond neurological status alone. Identifying which routinely calculated score best predicts seizures would allow clinicians to immediately risk-stratify patients without additional data collection. Third, the independent prognostic impact of PSS on clinical outcomes remains unclear. Multiple observational studies have consistently demonstrated that patients who develop seizures have substantially higher crude mortality than those who do not, leading to widespread perception that seizures directly worsen outcomes and justifying the use of prophylactic antiepileptic drugs in many centers (20, 21). However, patients who develop seizures also have more severe strokes, greater degrees of organ failure, and more comorbidities at baseline, raising the possibility that the observed mortality association is explained entirely by confounding by indication rather than a direct causal effect of seizures themselves (21). SeLECT-based studies have shown that acute symptomatic seizures carry high adjusted mortality risk (HR up to 14.2) (17), interpreted by some as evidence that seizures themselves precipitate death. However, these analyses rely on conventional Cox adjustment that may incompletely control for the strong confounding between stroke severity and seizure occurrence. Conversely, adjusted cohort studies have found no independent seizure mortality association after accounting for hemorrhage volume (22). However, critically ill ICU stroke patients differ substantially from general ward stroke populations in ways that may alter seizure epidemiology and detection. General ward-derived prediction models may therefore misestimate seizure risk in this population, underscoring the need for an ICU-specific tool.

To address these gaps, we conducted a large retrospective multicenter cohort study of 6,586 eligible critically ill stroke patients from 208 ICUs across the United States. Our study had three primary objectives: (1) to develop and internally validate a simple, readily applicable clinical prediction model for in-hospital PSS using only data routinely available within the first 24 h of ICU admission; (2) to perform a head-to-head comparison of the four most commonly used ICU severity scores for PSS prediction; and (3) to evaluate the independent impact of PSS on in-hospital mortality and length of stay using propensity score matching to minimize measured confounding. By delivering a zero-cost admission based risk tool, we aim to enable targeted EEG resource allocation and clarify whether PSS itself, not merely underlying stroke severity, should guide antiseizure management.

## Methods

### Study design and data source

We performed a retrospective multicenter cohort study using data from the eICU-Collaborative Research Database (eICU-CRD), a large, publicly available de-identified critical care database developed and maintained by the MIT Laboratory for Computational Physiology. The database contains high-quality granular data for over 200,000 ICU admissions across 208 hospitals throughout the United States between 2014 and 2015, including demographic information, vital signs, laboratory results, admission diagnoses, severity scores, treatments, and in-hospital outcomes. Hospital characteristics including bed size and US geographic region were summarized as potential sources of center-level heterogeneity (Supplementary Table 1). The database has been extensively validated for critical care research and has been used in hundreds of peer-reviewed publications across multiple critical care conditions. The Institutional Review Board of the Massachusetts Institute of Technology approved the creation of the database, and the requirement for informed consent was waived due to the complete de-identification of all patient data.

### Study population

We included all adult patients (≥18 years of age) admitted to the ICU with a primary diagnosis of acute stroke, defined using ICD-9430.xx (subarachnoid hemorrhage), 431.xx (intracerebral hemorrhage), 433.xx (occlusion of precerebral arteries), 434.xx (occlusion of cerebral arteries), and 436.xx (acute but ill-defined cerebrovascular disease); and ICD-10 codes I60.xx (subarachnoid hemorrhage), I61.xx (intracerebral hemorrhage), and I63.xx (cerebral infarction). We excluded patients with a documented history of epilepsy or pre-existing seizure disorder, those with ICU length of stay less than 24 h, patients with traumatic brain injury or primary brain tumor, and those with missing data for the primary outcome. Patients were followed from ICU admission until hospital discharge or death. We did not exclude patients with early mortality or withdrawal of care, as these represent important competing events in critical illness.

### Outcome and variables

The primary outcome for model development was coded in-hospital PSS, defined through ICD-9 codes 345.xx (epilepsy and recurrent seizures) and 780.3x (convulsions), and ICD-10 codes G40.xx (epilepsy) and R56.xx (convulsions, not elsewhere classified). We cannot distinguish whether a coded seizure was confirmed by routine EEG, continuous EEG, or clinical observation alone, as the eICU-CRD does not contain EEG modality, indication, or monitoring duration data. It is possible that sicker patients were more likely to undergo EEG monitoring, creating detection bias in which patients with more severe illness were more likely to have seizures documented. However, the eICU-CRD captures only events documented during the index hospitalization, and the exact time interval from stroke onset to ICU admission cannot be reliably derived from the database, as stroke onset time is not consistently recorded. Accordingly, our endpoint represents any seizure event coded during the ICU/hospitalization stay, which may include both early-onset (within 7 days) and later in-hospital seizures, and should be interpreted as coded in-hospital PSS rather than strictly time-defined acute symptomatic seizures. Consequently, the present model identifies factors associated with coded in-hospital PSS rather than predicting strictly incident events, because some coded seizures may have occurred before or at ICU admission and could not be temporally separated from first-24-h predictor measurement. We acknowledge that this endpoint captures only clinically recognized, administratively coded convulsive seizures; the eICU-CRD does not include standardized continuous EEG data, and therefore non-convulsive or subclinical electrographic seizures could not be identified. All model predictors were extracted from the first 24 h of ICU admission. Severity scores (APACHE II, SOFA, OASIS, GCS) are calculated from worst physiological values within this window; AKI (KDIGO criteria) and mechanical ventilation reflect the full ICU stay and were used solely as PSM matching covariates, not as predictors in the final model. For the prognostic analysis, the primary outcomes were all-cause in-hospital mortality and hospital length of stay.

We extracted all predictor variables from the first 24 h of ICU admission, including: demographic characteristics (age, sex, weight, height); admission severity scores (APACHE II, SOFA, Glasgow Coma Scale, OASIS); admission vital signs (heart rate, temperature, oxygen saturation); routine laboratory results (hemoglobin, white blood cell count, creatinine); and pre-existing comorbidities (diabetes mellitus, hypertension). We also extracted data on acute kidney injury (defined by KDIGO creatinine criteria) and receipt of mechanical ventilation. Stroke subtype was categorized as hemorrhagic (ICD-9430, 431; ICD-10 I60, I61), ischemic (ICD-9433, 434; ICD-10 I63), mixed (patients with both ischemic and hemorrhagic components, such as hemorrhagic transformation of cerebral infarction), or unspecified (ICD-9436, “acute but ill-defined cerebrovascular disease,” where imaging or clinical documentation did not distinguish ischemic from hemorrhagic pathology) based on primary diagnosis codes.

### Missing data handling

Variables with greater than 60% missing data were excluded from analysis entirely, as they could not be reliably imputed. For variables with 20–60% missingness, we used multiple imputation by chained equations (MICE) with 20 imputations. The imputation model included the outcome variable, all candidate predictors, and auxiliary variables associated with missingness. For variables with less than 20% missingness, we imputed missing values using the median for continuous variables and mode for categorical variables, as is standard in critical care prediction modeling. Missing indicator variables were not included in the final regression models. Missing proportions for all candidate predictors are summarized in Supplementary Table 2. We performed complete-case analysis as a sensitivity analysis to confirm the robustness of our results to missing data assumptions.

### Model development and validation

The eligible cohort was randomly divided into a training set and an internal validation set with a ratio of 7:3, stratified by seizure status. Variable selection followed a pre-specified three-step process. First, we performed univariate logistic regression in the training set for each candidate variable, retaining variables associated with PSS at *p* < 0.1 for further consideration; This univariate screening step was used solely to reduce the candidate variable set from a large number of routinely collected ICU variables to a more manageable number for LASSO, removing variables with no plausible univariate association before penalization; it does not alter the final variable selection, as LASSO performs the definitive shrinkage. Second, the retained variables were entered into least absolute shrinkage and selection operator (LASSO) regression with 10-fold cross-validation. Third, all variables with non-zero coefficients in the LASSO model were entered into multivariable logistic regression, and variables with two-sided *p* < 0.05 were retained in the final model. After LASSO identified variables with non-zero coefficients, these were entered into a standard multivariable logistic regression without penalization, and coefficients were re-estimated by maximum likelihood. All preprocessing steps including univariate screening, LASSO selection, and coefficient estimation were performed exclusively within the training set; the validation set was used only for performance evaluation. Model discrimination was assessed using the area under the receiver operating characteristic curve (ROC). Model calibration was evaluated using calibration plots and the calibration slope. Overall model fit was assessed using the Brier score. Clinical utility was evaluated using decision curve analysis. We performed bootstrap validation with 500 resamples to obtain bias-corrected 95% confidence intervals for all performance metrics.

The final model included five predictors and 493 PSS events, yielding an events-per-variable ratio of 98.6, well above the commonly recommended minimum of 10 events per variable for logistic regression prediction models. This study was conducted and reported in accordance with the TRIPOD (Transparent Reporting of a multivariable prediction model for Individual Prognosis Or Diagnosis) guidelines.

### Propensity score matched prognostic analysis

To minimize confounding when evaluating the prognostic impact of PSS on mortality, we performed propensity score matching. We calculated propensity scores using multivariable logistic regression including confounders: age, sex, APACHE II score, SOFA score, GCS, diabetes mellitus, hypertension, acute kidney injury, mechanical ventilation, and stroke subtype (hemorrhagic vs. ischemic). We then performed 1:1 nearest-neighbor matching without replacement, using a caliper of 0.05 standard deviations of the logit propensity score to ensure close matches. Covariate balance after matching was assessed using standardized mean differences (SMD), with SMD < 0.1 considered to indicate excellent balance with negligible residual confounding. Covariate balance before and after matching for all matched variables is presented in Supplementary Table 3. After matching, we used conditional logistic regression to estimate the matched adjusted odds ratios for in-hospital mortality, accounting for the paired structure, and paired linear regression for differences in length of stay. Given the expected right-skewed distribution of hospital length of stay, median (IQR) values are additionally reported, and the Wilcoxon signed-rank test was used as a non-parametric sensitivity analysis for the matched comparison.

### Statistical analysis

Continuous variables are presented as median (interquartile range) due to non-normal distributions, and were compared between groups using the Mann–Whitney U test. Categorical variables are presented as counts (percentages) and compared using chi-square tests or Fisher’s exact tests as appropriate. All statistical tests were two-sided, with *p* < 0.05 considered statistically significant. All analyses were performed using R version 4.2.0.

## Results

### Baseline characteristics of the study cohort

We initially identified 9,248 adult patients admitted to 208 ICUs in the eICU-CRD between 2014 and 2015 with a primary discharge diagnosis of acute stroke (Supplementary Figure 1). Finally, a total of 6,586 stroke patients were enrolled in the study, consisting of 493 patients with in-hospital PSS and 6,093 patients without PSS events. Seizure patients had a lower median age [67.00 (IQR 55.00–76.00)] than non-seizure patients [69.00 (IQR 57.00–79.00)] (*p* = 0.002). Seizure patients presented higher median body temperature and heart rate (*p* < 0.001), while respiratory rate was similar (*p* = 0.727). Median non-invasive mean and systolic blood pressure were lower in seizure patients (*p* = 0.035 and *p* = 0.003, respectively), with no difference in diastolic blood pressure (*p* = 0.774). All illness severity scores (APACHE, APS, OASIS, SOFA) were significantly higher in seizure patients (*p* < 0.001), whereas the median GCS score was markedly lower in this group [13.00 (IQR 9.00–15.00) vs. 15.00 (IQR 13.00–15.00), *p* < 0.001].

For laboratory parameters, seizure patients had lower median hemoglobin (*p* < 0.001) and slightly elevated creatinine (*p* = 0.018) and INR (*p* = 0.010). Glucose, blood urea nitrogen, platelet count, and white blood cell count did not differ significantly between groups (*p* > 0.05).

For comorbidity and organ support, the seizure group presented higher prevalence rates of diabetes mellitus (20.89% vs. 12.67%, *p* < 0.001), hypertension (72.62% vs. 67.91%, *p* = 0.031), acute kidney injury (71.60% vs. 60.56%, *p* < 0.001), and invasive mechanical ventilation use (16.02% vs. 6.37%, *p* < 0.001).

Stroke subtype distribution varied significantly across the two cohorts (*p* = 0.019). Hemorrhagic stroke accounted for a larger proportion of seizure patients (41.99%) relative to non-seizure patients (36.21%), whereas ischemic stroke was more prevalent in the non-seizure group (31.33% vs. 25.15%). BMI stratification revealed a U-shaped association with seizure risk: underweight patients (BMI < 18.5) exhibited the highest seizure rate at 13.48% (31/230), followed by normal weight patients (7.72%, 149/1930) and obese patients (7.52%, 170/2261), while overweight patients had the lowest seizure rate at 6.58% (141/2144), reflecting an approximately 2-fold difference in seizure risk between underweight and overweight patients. Although a U-shaped association between BMI categories and PSS incidence was observed in the baseline descriptive analysis, BMI entered as a continuous variable did not meet the pre-specified *p* < 0.1 threshold in univariable logistic regression (OR = 1.000, 95%CI 0.979–1.002, *p* = 0.958), and its coefficient was shrunk to zero by LASSO regularization at the lambda.min criterion. Accordingly, BMI was not retained in the final multivariable model. The U-shaped BMI-PSS association should be interpreted as a post-hoc exploratory finding, not as a validated predictor, and requires external confirmation before any clinical application. In post-hoc sensitivity analyses, a quadratic BMI term significantly improved model fit (likelihood-ratio test *p* = 0.012), and categorical logistic regression (normal weight as reference) confirmed a higher adjusted PSS risk in underweight patients (OR = 1.86, 95%CI 1.23–2.82, *p* = 0.003), whereas overweight (OR = 0.84, 95%CI 0.66–1.07) and obese (OR = 0.97, 95%CI 0.77–1.22) categories did not differ significantly. As the pre-specified pipeline entered BMI only as a linear term, which has limited power to capture non-linear effects, the U-shaped association remains hypothesis-generating and requires prospective confirmation.

Median hospitalization duration was longer in patients with seizures [8.00 (IQR 5.00–14.00) days vs. 6.00 (IQR 4.00–11.00) days, *p* < 0.001]. In-hospital mortality was significantly higher among seizure patients (17.65% vs. 12.82%, *p* = 0.002; Table 1).

**Table 1:** Demographic, clinical, laboratory and outcome characteristics of the study cohort stratified by seizure status.

Characteristics	No PSS (*n* = 6,093)	PSS (*n* = 493)	*p*
Age, years	69.00 (57.00, 79.00)	67.00 (55.00, 76.00)	0.002
Gender, *n* (%)			0.548
Female	2,940 (48.25)	230 (46.65)	
Male	3,153 (51.75)	263 (53.35)	
BMI, kg/m^2^	27.45 (23.82, 31.95)	26.75 (23.03, 31.87)	0.063
Respiratory rate, breaths/min	18.00 (15.00, 22.00)	18.00 (16.00, 22.00)	0.727
Temperature, °C	36.70 (36.40, 37.00)	36.80 (36.50, 37.20)	<0.001
Heart rate, beats/min	80.00 (69.00, 93.00)	83.00 (72.00, 96.00)	<0.001
NIBP diastolic, mmHg	74.00 (63.00, 86.00)	73.00 (62.00, 88.00)	0.774
NIBP mean, mmHg	92.73 (80.00, 106.00)	90.00 (76.00, 105.00)	0.035
NIBP systolic, mmHg	140.00 (123.00, 158.00)	137.00 (116.00, 156.00)	0.003
APACHE II score	51.07 (38.00, 68.00)	60.00 (45.00, 81.00)	<0.001
APS score	36.35 (26.00, 53.00)	48.00 (33.00, 68.00)	<0.001
GCS score	15.00 (13.00, 15.00)	13.00 (9.00, 15.00)	<0.001
OASIS score	27.00 (22.00, 35.00)	33.00 (25.00, 40.00)	<0.001
SOFA score	3.00 (1.00, 5.00)	5.00 (3.00, 7.00)	<0.001
Glucose, mg/dL	132.00 (104.00, 179.05)	135.00 (104.00, 188.00)	0.135
BUN, mg/dL	17.00 (13.00, 24.00)	18.20 (12.00, 28.00)	0.097
Creatinine, mg/dL	0.94 (0.73, 1.26)	1.00 (0.71, 1.50)	0.018
Hgb, g/dL	12.90 (11.30, 14.30)	12.40 (10.60, 14.00)	<0.001
Platelet count, ×10^9^/L	213.00 (172.00, 264.00)	211.00 (159.00, 273.00)	0.198
WBC, ×10^9^/L	9.60 (7.40, 12.70)	9.80 (7.40, 13.50)	0.327
INR	1.10 (1.00, 1.30)	1.10 (1.00, 1.43)	0.010
Hospitalization duration, days	6.00 (4.00, 11.00)	8.00 (5.00, 14.00)	<0.001
Discharge status, *n* (%)			0.002
Alive	5,312 (87.18)	406 (82.35)	
Expired	781 (12.82)	87 (17.65)	
Stroke type, *n* (%)			0.019
Hemorrhagic	2,206 (36.21)	207 (41.99)	
Ischemic	1909 (31.33)	124 (25.15)	
Mixed	121 (1.99)	9 (1.83)	
Unspecified	1857 (30.48)	153 (31.03)	
BMI group, *n* (%)			0.002
Underweight (<18.5)	199 (3.28)	31 (6.31)	
Normal (18.5–25)	1781 (29.32)	149 (30.35)	
Overweight (25–30)	2003 (32.98)	141 (28.72)	
Obese (≥30)	2091 (34.43)	170 (34.62)	
DM, *n* (%)			<0.001
No	5,321 (87.33)	390 (79.11)	
Yes	772 (12.67)	103 (20.89)	
Hypertension, *n* (%)			0.031
No	1955 (32.09)	135 (27.38)	
Yes	4,138 (67.91)	358 (72.62)	
AKI, *n* (%)			<0.001
No	2,403 (39.44)	140 (28.40)	
Yes	3,690 (60.56)	353 (71.60)	
Ventilation, *n* (%)			<0.001
No	5,705 (93.63)	414 (83.98)	
Yes	388 (6.37)	79 (16.02)	

APS, Acute Physiology Score; APACHE II, Acute Physiology and Chronic Health Evaluation II; OASIS, Oxford Acute Severity of Illness Score; SOFA, Sequential Organ Failure Assessment; GCS, Glasgow Coma Scale; BMI, body mass index; INR, international normalized ratio; WBC, white blood cell; BUN, blood urea nitrogen; AKI, acute kidney injury; ICU, intensive care unit; PSS, post-stroke seizure; IQR, interquartile range. Data are presented as median (IQR) for continuous variables and n (%) for categorical variables. Group comparisons: continuous variables were compared using the Mann–Whitney U test; categorical variables using the chi-square test or Fisher’s exact test as appropriate.

### Comparison of routine ICU severity scores for PSS prediction

In head-to-head comparison of the four routine ICU severity scores for PSS prediction in the entire cohort, SOFA score demonstrated the best discriminative performance with an AUC of 0.683 (95% CI 0.662–0.703), followed by GCS (AUC = 0.646, 95% CI 0.625–0.666), OASIS (AUC = 0.636, 95% CI 0.613–0.659), and APACHE II score which had the lowest discriminative ability (AUC = 0.611, 95% CI 0.588–0.634) (Figure 1). DeLong’s test confirmed that SOFA significantly outperformed GCS (*z* = 5.02, *p* < 0.0001), OASIS (*p* < 0.001), and APACHE II (*p* < 0.0001). To determine whether SOFA’s superior performance was driven primarily by its neurological (GCS) component, we decomposed the SOFA score. The GCS subscore alone yielded an AUC of 0.616, while the remaining five non-neurological SOFA components achieved an AUC of 0.627. The correlation between total SOFA and GCS was modest (*r* = −0.57). These findings indicate that SOFA’s discriminative performance reflects not merely its neurological component, but also incremental information from systemic organ dysfunction across non-neurological organ systems.

**Figure 1:**
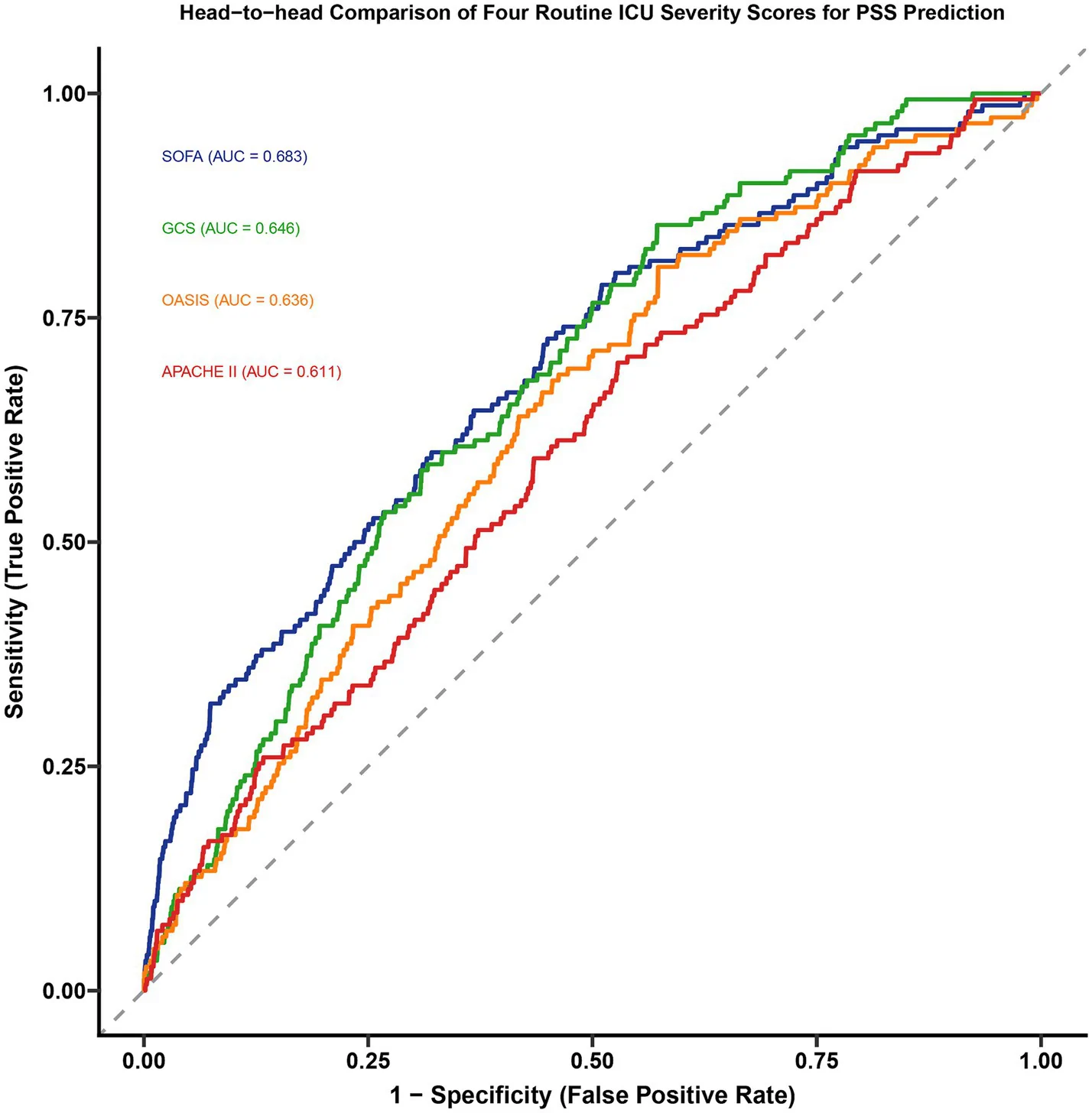
Receiver operating characteristic (ROC) curves comparing discrimination performance of four routine ICU severity scores for prediction of in-hospital PSS. SOFA, Sequential Organ Failure Assessment; GCS, Glasgow Coma Scale; OASIS, Oxford Acute Severity of Illness Score; APACHE II, Acute Physiology and Chronic Health Evaluation II; AUC, area under the receiver operating characteristic curve; PSS, post-stroke seizure.

### Independent predictors and model performance

All candidate predictors were initially screened through univariable logistic regression (Supplementary Table 4), and variables with *p* < 0.1 were incorporated into LASSO regression with 10-fold cross-validation using the lambda.min criterion to eliminate redundant predictors (Supplementary Figure 2). Subsequent multivariable logistic regression identified five independent risk factors for PSS: age (adjusted OR = 0.987, 95%CI 0.980–0.994, *p* < 0.001), admission temperature (adjusted OR = 1.224, 95%CI 1.041–1.440, *p* = 0.015), hypertension history (adjusted OR = 1.354, 95%CI 1.051–1.758, *p* = 0.021), mean corpuscular hemoglobin concentration (MCHC) (adjusted OR = 0.898, 95%CI 0.829–0.973, *p* = 0.008), and GCS score (adjusted OR = 0.793, 95%CI 0.681–0.923, *p* = 0.003). The logit prediction equation of the final model was constructed as follows:


$$\begin{array}{l} \text{Logit}(P)=\beta_{0}-0.013\times \mathrm{age}+0.202\times \text{temperature}\\ +0.304\times \text{Hypertension}-0.108\times \text{MCHC}-0.232\times \mathrm{GCS} \end{array}$$

The full model coefficients and performance metrics are summarized in Supplementary Table 5.

Given that MCHC may be influenced by ICU-specific pre-analytical factors including intravenous fluid resuscitation, blood transfusion, and sample hemolysis, we performed several post-hoc sensitivity analyses on the final 6,586-patient cohort. After excluding 441 patients with hemoglobin <9 g/dL (*n* = 6,145 retained), lower MCHC remained an independent predictor of coded PSS (adjusted OR = 0.879, 95%CI 0.816–0.946, *p* < 0.001). After excluding 101 patients with MCHC <30 g/dL (*n* = 6,485 retained), the association remained significant (adjusted OR = 0.883, 95%CI 0.819–0.953, *p* = 0.001). In a combined analysis excluding both groups (*n* = 6,082 retained), MCHC retained its independent association (adjusted OR = 0.877, 95%CI 0.808–0.951, *p* = 0.002).

Variance inflation factor analysis ruled out obvious multicollinearity among these predictors. Pearson correlation matrix heatmap of the five independent predictors was displayed in Supplementary Figure 3. The model showed moderate and stable discrimination, with an AUC of 0.702 (95% CI 0.665–0.739) in the training set and 0.713 (95% CI 0.668–0.758) in the internal validation set (Figure 2A) (optimism-corrected AUC after 500 bootstrap resamples: 0.694). Calibration was adequate across training (H-L *p* = 0.678, Brier = 0.066, calibration intercept = 0.04, calibration slope = 0.98) and validation (H-L *p* = 0.565, Brier = 0.074, calibration intercept = 0.12, calibration slope = 1.03) cohorts (Figures 2B,C). DCA confirmed robust clinical utility, with positive net benefit at risk thresholds of 3–18% in both datasets (Figures 2D,E).

**Figure 2:**
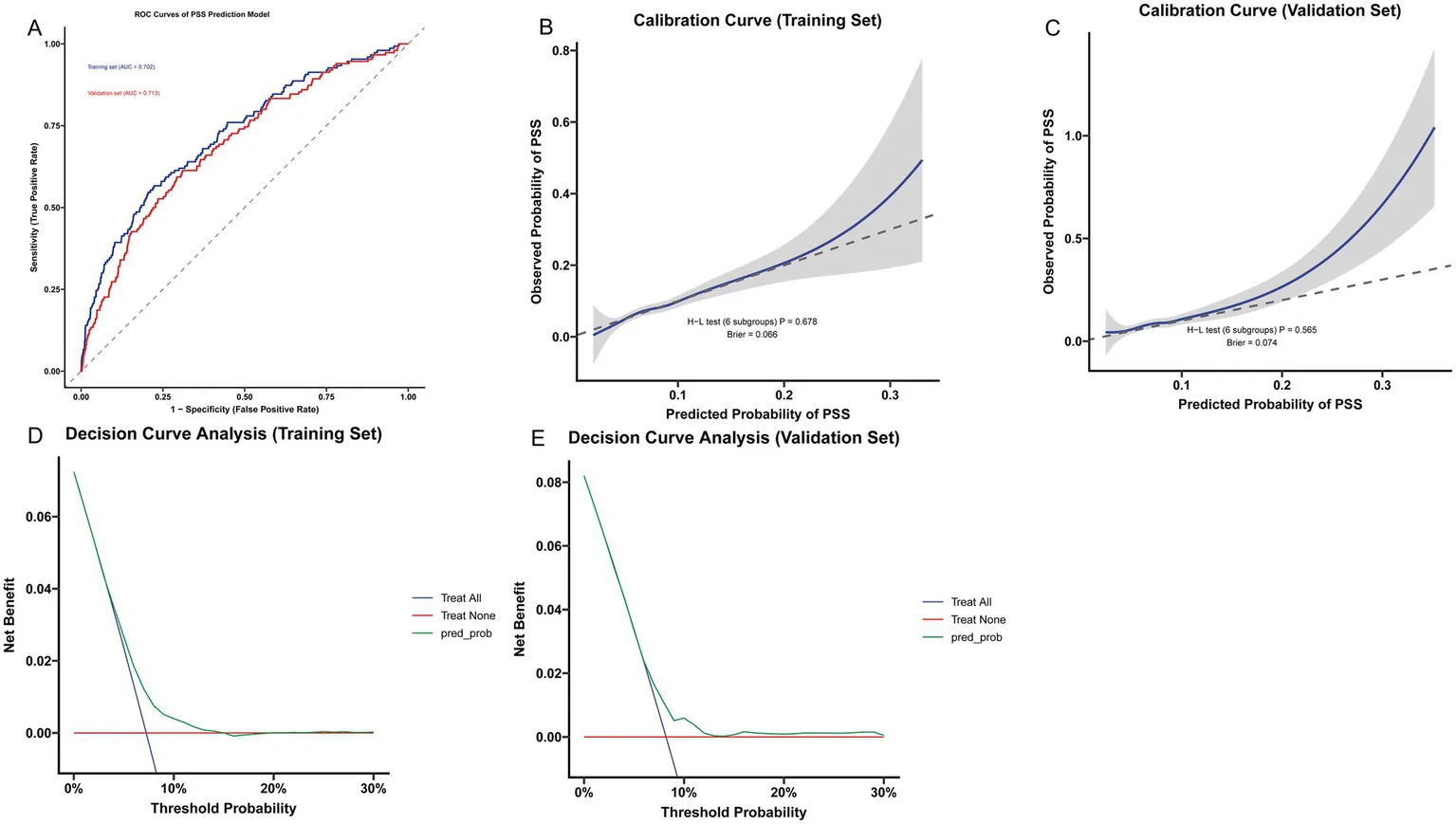
Comprehensive performance evaluation of the logistic prediction model for PSS. **(A)**, ROC curves for the training and internal validation cohorts; **(B)**, Calibration curve of the model in the training set; **(C)**, Calibration curve of the model in the validation set; **(D)**, Decision curve analysis (DCA) for the training cohort; **(E)**, DCA for the validation cohort. AUC, area under the receiver operating characteristic curve; H-L, Hosmer–Lemeshow; DCA, decision curve analysis; PSS, post-stroke seizure.

### Subgroup and sensitivity analyses

Model performance was generally consistent across all clinical subgroups. Discrimination was similar in younger [<65 years, *N* = 2,685, events = 214, ROC = 0.72 (95% CI 0.68–0.76)] and older [≥65 years, *N* = 3,901, events = 279, ROC = 0.70 (0.66–0.74)] patients (*p* for interaction = 0.12), in males [*N* = 3,480, events = 255, ROC = 0.71 (0.67–0.75)] and females [*N* = 3,106, events = 238, ROC = 0.73 (0.69–0.77); *P* for interaction = 0.55], and numerically higher in patients with hemorrhagic stroke [*N* = 2,413, events = 207, ROC = 0.71 (0.67–0.75)] compared to those with ischemic stroke [*N* = 2033, events = 124, ROC = 0.70 (0.65–0.75); *p* for interaction = 0.41; Figure 3].

**Figure 3:**
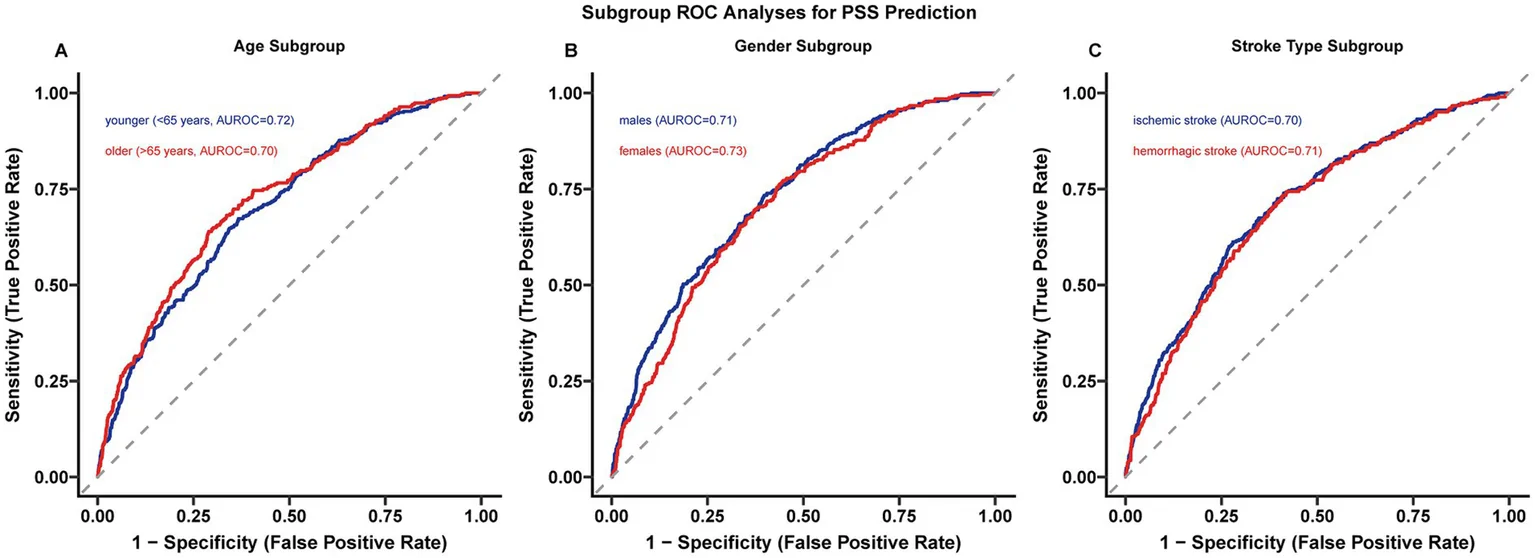
Subgroup ROC curve analyses for PSS prediction in the full cohort stratified by age **(A)**, gender **(B)** and stroke subtype **(C)**.

Multiple sensitivity analyses confirmed the robustness of our findings: excluding patients who received mechanical ventilation resulted in ROC of 0.74, complete-case analysis without imputation yielded nearly identical performance (ROC = 0.70) (Supplementary Figure 4). A complete-case analysis excluding all imputed observations yielded effect estimates consistent with the primary imputed analysis (complete-case ORs: age 0.988, temperature 1.126, hypertension 1.294, MCHC 0.872, GCS 0.873), with all five predictors retaining the same direction and statistical significance.

As a sensitivity analysis, we repeated model development after excluding patients with unspecified stroke subtype. Model discrimination remained moderate (training AUC = 0.698, validation AUC = 0.705), and all five predictors retained consistent effect directions and statistical significance, indicating that the unspecified subgroup did not materially influence model findings.

Detailed results of all sensitivity analyses are summarized in Supplementary Table 6.

### Clinical risk stratification

Based on the distribution of predicted probabilities, we developed a simple three-tier risk stratification scheme for clinical use. Thresholds of 5 and 10% were chosen *a priori* based on clinical interpretability: the 5% threshold approximates the overall cohort incidence (7.5%), while the 10% threshold identifies patients at approximately twice the average risk. Patients with predicted risk <5% were categorized as low risk (*N* = 1763, observed PSS rate 3.4%), those with predicted risk 5–10% as intermediate risk (*N* = 3,692, observed rate 6.8%), and those with predicted risk >10% as high risk (*N* = 1,131, observed rate 16.2%; Figure 4). Consistent risk stratification was observed in both cohorts: in the training set, the low, intermediate, and high risk groups comprised 1,227 (46 events, 3.7%), 2,588 (172 events, 6.6%), and 795 patients (127 events, 16.0%), respectively; in the validation set, corresponding numbers were 536 (14 events, 2.6%), 1,104 (78 events, 7.1%), and 336 patients (56 events, 16.7%). At the high-risk threshold (>10%), the model achieved a sensitivity of 37%, specificity of 84%, positive predictive value of 16%, and negative predictive value of 94%. Reproducible rules for the three-tier risk stratification scheme are as follows: low risk (predicted *p* < 5%), intermediate risk (predicted *P* 5–10%), and high risk (predicted *p* > 10%). A simplified bedside scoring tool derived from the regression coefficients is provided in Supplementary Table 7 to facilitate rapid risk screening without calculator-based computation. Patients in the high-risk stratum had a 4.8-fold higher rate of PSS compared to low-risk patients.

**Figure 4:**
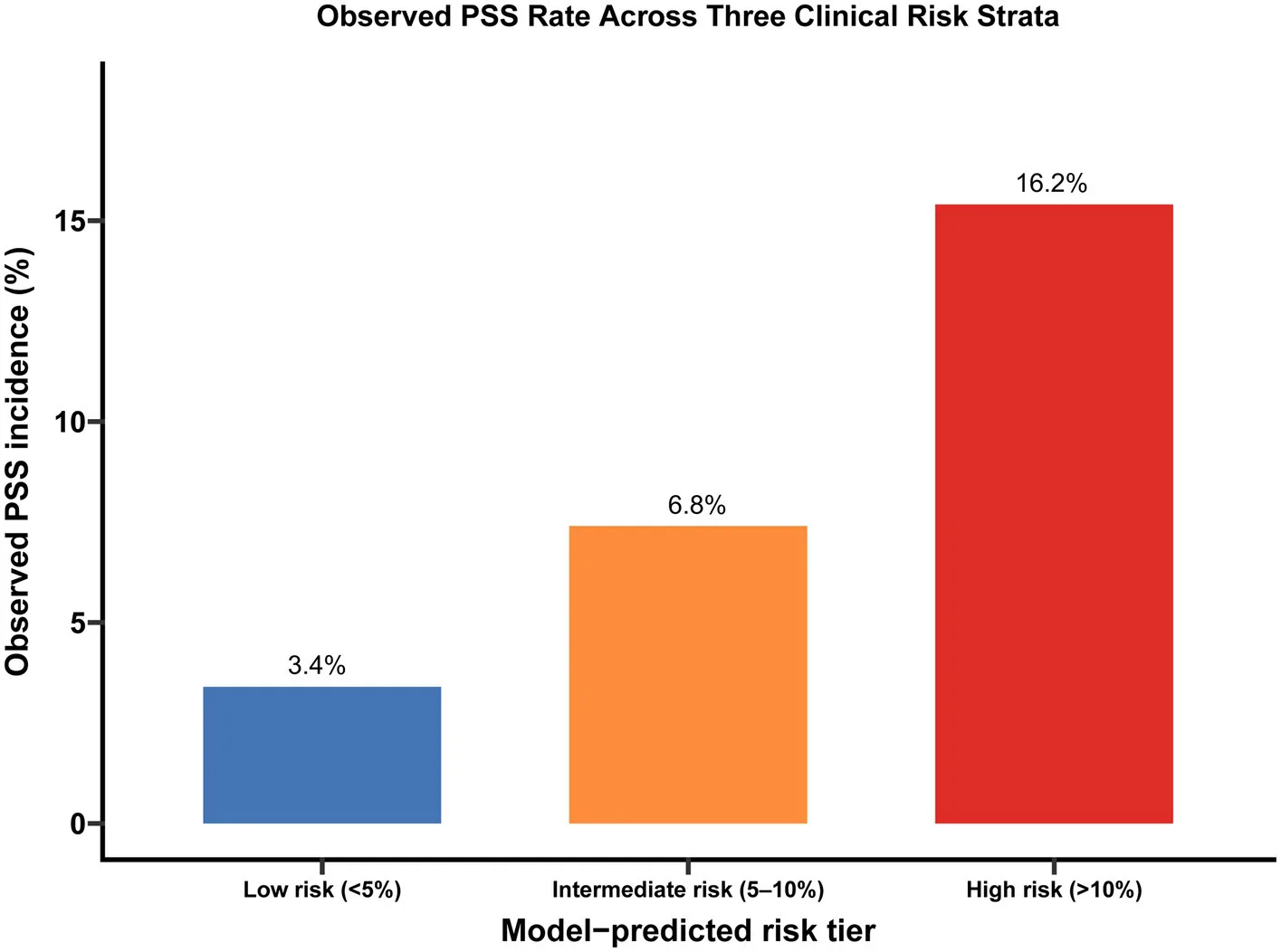
Observed incidence of PSS across three risk tiers stratified by model-predicted probability.

### Prognostic impact of PSS after propensity score matching

After matching, 493 patients with PSS were matched to 493 controls. All standardized mean differences were below 0.10 after matching, indicating excellent covariate balance (Supplementary Table 3). The higher crude mortality in the PSS group was attenuated, with slightly higher non-significant mortality in matched controls, reflecting elimination of baseline severity confounding. There was no statistically significant difference in in-hospital mortality between groups: mortality was 18.1% in patients with PSS compared to 20.2% in matched controls, corresponding to an adjusted odds ratio of 0.84 (95% CI 0.62–1.16, *p* = 0.295). Similarly, there was no significant difference in mean hospital length of stay between groups (11.5 days in PSS vs. 11.8 days in controls, 95% CI -0.72–0.12, *p* = 0.167; Figure 5), with comparable median values [median 8 days (IQR 5–14) in PSS vs. 8 days (IQR 4–13) in matched controls; Wilcoxon signed-rank *p* = 0.21]. We acknowledge that death during hospitalization represents a competing risk that may shorten observed length of stay; however, mortality rates did not differ significantly between matched groups, limiting the potential impact of this competing event on the LOS comparison.

**Figure 5:**
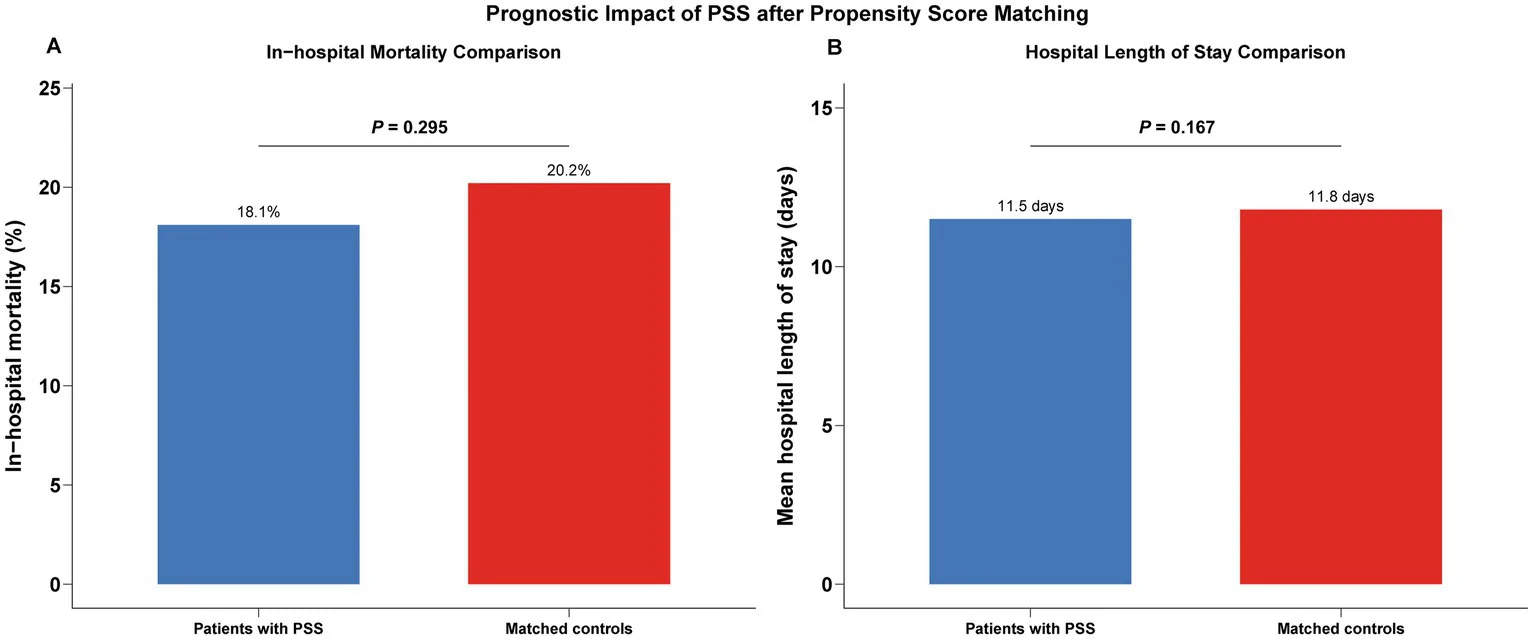
Comparison of short-term in-hospital outcomes between patients with PSS and matched controls after PSM. **(A)**, Bar chart showing the proportion of in-hospital mortality. **(B)**, Bar chart illustrating the mean hospital length of stay. PSS, post-stroke seizure; PSM, propensity score matching.

## Discussion

This multicenter retrospective cohort study of 6,586 critically ill stroke patients developed and internally validated a prediction model for coded in-hospital PSS, systematically compared the discriminative performance of four routine ICU severity scores for coded PSS risk stratification, and clarified the independent short-term prognostic impact of PSS. The final model incorporated 5 routinely available admission variables with stable discrimination across training and validation cohort. We emphasize that the endpoint captured in this study reflects clinically evident, administratively coded convulsive seizures; non-convulsive and electrographic seizures, which are highly prevalent in ICU stroke populations but require cEEG for detection, were not captured and are outside the scope of this model.

Of the five independent predictors identified in our final model, four are consistent with established pathophysiological mechanisms and replicate findings from existing PSS prediction cohorts.

Younger age was associated with higher PSS risk, aligning with the international IsCHEMiA imaging score validation study which identified age <65 years as a core predictor of post-stroke epilepsy (23). This likely reflects higher cortical excitability and more robust inflammatory responses to acute brain injury in younger patients, in contrast to older patients with age-related cortical atrophy that provides more space for cerebral edema and reduces cortical irritation (24). Elevated admission temperature independently predicted PSS, consistent with experimental and clinical data demonstrating that pyrexia accelerates glutamate excitotoxicity, increases blood–brain barrier permeability, and amplifies neuroinflammatory cascades that acutely lower seizure threshold (25). Pre-existing hypertension increased PSS odds by 35%, replicating findings from a recent multicenter Saudi Arabian cohort which identified chronic hypertension as a robust PSS risk factor, likely mediated by longstanding hypertensive small vessel disease, increased risk of hemorrhagic transformation, and chronic endothelial dysfunction that primes the cortex for ictal activity (26). Lower GCS score, reflecting more severe global neurological injury and cortical involvement, was a consistent predictor across all published PSS models including SeLECT-EEG and post-thrombectomy machine learning scores, as depressed consciousness correlates with larger lesion volume, mass effect, and midline shift (27, 28). Notably, lower MCHC emerged as a novel independent predictor not included in any of the currently widely used PSS risk scores and this association remained robust across multiple sensitivity analyses. We interpret this finding as hypothesis-generating rather than establishing a definitive mechanism. MCHC reflects erythrocyte hemoglobin concentration (hypochromia) rather than total hemoglobin mass or systemic oxygen-carrying capacity, and therefore the cerebral oxygen delivery hypothesis should be regarded as one of several possible explanations. Lower MCHC may alternatively act as a marker of chronic systemic disease, subclinical inflammation, iron or nutritional deficiency, or disordered erythropoiesis, all of which are plausibly linked to both reduced physiological reserve and altered neuronal excitability after stroke. This finding complements recently reported “double-hit” models of post-stroke epileptogenesis that integrate hypoxic-metabolic stress alongside structural brain injury (29), but requires external replication and mechanistic studies before causal inference. Critically, unlike existing high-performance models that require neuroimaging data on cortical involvement (23), continuous EEG biomarkers (27), or complex machine learning pipelines (28, 30), our model relies exclusively on variables available within minutes of ICU admission. In contrast to SeLECT, IsCHEMiA, and SeLECT-EEG, which require imaging or EEG data unavailable in the first ICU day and were derived in ward cohorts, our model uses five variables available within 24 h, trading modestly lower AUC for universal bedside availability. The superior discriminative performance of SOFA compared to GCS, APACHE II, and OASIS carries important conceptual implications for PSS pathophysiology. While GCS only captures neurological dysfunction, SOFA integrates dysfunction across six organ systems, and decomposition analysis showed that its advantage was not driven solely by the embedded GCS component (non-neurological SOFA components achieved AUC = 0.627), supporting a contribution of systemic organ dysfunction beyond neurological status alone. However, the absolute incremental gain of SOFA over the simpler GCS was modest (AUC 0.683 vs. 0.646), and both scores are already calculated routinely at ICU admission with no additional data collection burden. We therefore do not suggest that SOFA should replace neurological assessment; rather, the modest but statistically significant advantage indicates that systemic physiological derangement carries complementary signal, and clinicians may prioritize SOFA when multi-organ severity is being summarized, while GCS remains a rapid standalone neurological screen.

The DCA results provide additional context for clinical application. The model’s positive net benefit across the 3–18% threshold range suggests that it is most valuable for clinicians whose practice threshold for escalating neurological monitoring falls within this range. In practical terms, for a patient with a predicted PSS risk >10% (high-risk tier), the model supports escalation to continuous EEG monitoring and more frequent neurological assessment, as the expected benefit of seizure detection outweighs the cost of monitoring. In practice, the model can be computed at ICU admission from routine data to prioritize high-risk patients for limited cEEG resources and intensified neurological observation, while low-risk patients may avoid unnecessary monitoring; this tiered allocation requires prospective workflow evaluation. For patients in the 5–10% intermediate-risk tier, the model may support shared decision-making regarding enhanced monitoring, particularly in the presence of other clinical risk factors. For patients with predicted risk <3%, the model does not support additional monitoring beyond standard care. We emphasize that these threshold-based interpretations are research-oriented and require external validation before incorporation into clinical protocols.

While multiple cohorts have reported 2–3 fold higher mortality in patients with PSS, leading to widespread off-label use of prophylactic antiepileptic drugs out of concern for excess death, our analysis found no statistically significant independent association between PSS and in-hospital mortality or length of stay, although the wide confidence interval for mortality (0.62–1.16) cannot exclude a clinically meaningful effect and residual confounding remains possible, consistent with a smaller population-based study that also found no excess mortality after adjusting for baseline stroke severity (22). A systematic review noted substantial heterogeneity in PSS outcome estimates, with effect sizes attenuating dramatically as more confounders were adjusted for (21). Our data extend these findings by demonstrating that after balancing age, comorbidities, organ failure severity, and neurological status, PSS is a marker of underlying illness severity rather than a causal mediator of death. We emphasize that this analysis is limited to demonstrating the absence of an independent prognostic effect of coded PSS per se on short-term in-hospital mortality, and cannot resolve the broader clinical question of antiseizure prophylaxis. Specifically, our study does not address whether prophylactic antiseizure medication yields net clinical benefit in high-risk patients, nor does it refute the established therapeutic role of antiseizure drugs for patients who have already experienced a clinical seizure. These results do not support routine, prolonged prophylactic antiepileptic drug administration for all critically ill stroke patients for the purpose of reducing mortality.

Stratified analyses across BMI categories and stroke subtypes revealed clinically meaningful heterogeneity in PSS risk that has been underappreciated in prior cohorts. We observed a clear U-shaped association between BMI and PSS incidence, with the highest rate (13.5%) in underweight patients and the lowest rate (6.5%) in overweight patients, extending the well-documented neurological obesity paradox from stroke mortality to post-stroke seizure risk (31–33). Underweight patients frequently have chronic malnutrition, reduced systemic metabolic reserve, and heightened baseline inflammatory tone that increases neuronal vulnerability to ictal activity (34, 35), while mild overweight status provides nutritional and metabolic buffering against acute excitotoxic and inflammatory stress after stroke, mirroring similar risk gradients observed for post-stroke mortality and poor functional outcome (36). This exploratory association may partly reflect confounding or reverse causation by chronic illness, frailty, and nutritional status, and warrants prospective confirmation.

Across stroke subtypes, PSS rates were slightly higher in patients with hemorrhagic stroke compared to ischemic stroke, consistent with the known irritant effect of extravasated blood on cerebral cortex (8, 37), though model discrimination remained stable (AUC 0.71 vs. 0.70) in both subgroups, confirming that the 5-variable model performs consistently regardless of stroke pathophysiology. The elevated PSS risk in underweight patients in particular represents a simple, easily identifiable high-risk group that should be prioritized for closer neurological monitoring and lower threshold for continuous EEG, even in the absence of other traditional PSS risk factors.

Our study has several important limitations that should be acknowledged when interpreting our results. First, this retrospective analysis of a single US database (2014–2015) limits generalizability, and variation in detection and treatment across the 208 ICUs may introduce center-level heterogeneity. Second, despite adjustment and PSM, residual confounding remains: lesion characteristics, EEG abnormalities, post-admission complications, and antiseizure medications were not captured, and the 7.5% incidence may underestimate the true burden. Third, follow-up was limited to hospitalization, precluding assessment of late seizures and post-discharge outcomes. Fourth, the model was only internally validated. Fifth, ICD-based ascertainment without standardized cEEG captures only clinically evident coded convulsive seizures, and the temporal relationship between predictors and seizure onset cannot be established. Nearly one-third of patients lacked a specified stroke subtype, although a sensitivity analysis excluding them yielded consistent findings.

## Conclusion

In this multicenter cohort of critically ill stroke patients, we developed and internally validated a simple five-variable model for coded in-hospital PSS using routine first-24-h data; given its moderate discrimination, it is intended for risk stratification rather than individual prediction. SOFA outperformed the other routine ICU severity scores. After PSM, coded PSS showed no statistically significant association with in-hospital mortality, although the wide confidence interval and residual confounding preclude excluding a clinically meaningful effect. As this retrospective study relied on ICD coding without cEEG, findings apply only to clinically evident coded convulsive seizures. External validation and prospective evaluation with standardized cEEG are required before clinical implementation.

## Data Availability

The original contributions presented in the study are included in the article/Supplementary material, further inquiries can be directed to the corresponding author.
