# Supplementary material for: Development and validation of a simple clinical prediction model for post-stroke seizures in critically ill patients: a multicenter retrospective cohort study
Source: Front Neurol. 2026 Oct 13;17:1949946. doi: 10.3389/fneur.2026.1949946 (PMC13710065; doi:10.3389/fneur.2026.1949946)
Supplement: Supplementary file 1 [file Table_5.docx]

| Variable | β | OR | 95% CI | *P*-value |
| --- | --- | --- | --- | --- |
| Intercept | −5.83 | - | - | - |
| Age (per year) | −0.013 | 0.987 | 0.980–0.994 | <0.001 |
| Temperature (per °C) | 0.202 | 1.224 | 1.041–1.440 | 0.015 |
| Hypertension (yes vs. no) | 0.304 | 1.354 | 1.051–1.758 | 0.021 |
| MCHC (per g/dL) | −0.108 | 0.898 | 0.829–0.973 | 0.008 |
| GCS (per point) | −0.232 | 0.793 | 0.681–0.923 | 0.003 |
| Performance metrics |  |  |  |  |
| AUROC (95% CI) |  |  | Training: 0.702 (0.665–0.739) | Validation: 0.713 (0.668–0.758) |
| Optimism-corrected AUROC |  |  | 0.694 | - |
| Brier score |  |  | 0.066 | 0.074 |
| Calibration intercept |  |  | 0.04 | 0.12 |
| Calibration slope |  |  | 0.98 | 1.03 |
| Hosmer-Lemeshow P |  |  | 0.678 | 0.565 |

Supplementary Table 5 Final multivariable logistic regression model and performance metrics Notes: β, regression coefficient; OR, odds ratio; CI, confidence interval; MCHC, mean corpuscular hemoglobin concentration; GCS, Glasgow Coma Scale; AUROC, area under the receiver operating characteristic curve.
