# Supplementary material for: Development and validation of a simple clinical prediction model for post-stroke seizures in critically ill patients: a multicenter retrospective cohort study
Source: Front Neurol. 2026 Oct 13;17:1949946. doi: 10.3389/fneur.2026.1949946 (PMC13710065; doi:10.3389/fneur.2026.1949946)
Supplement: Supplementary file 2 [file Table_6.docx]

| Analysis | N | PSS n(%) | Age OR | Temp OR | HTN OR | MCHC OR | GCS OR | Train AUC | Val AUC |
| --- | --- | --- | --- | --- | --- | --- | --- | --- | --- |
| Primary (imputed) | 6586 | 493 (7.5) | 0.987 | 1.224 | 1.354 | 0.898 | 0.793 | 0.702 | 0.713 |
| Complete-case | 5420 | 410 | 0.988 | 1.126 | 1.294 | 0.872 | 0.873 | 0.700 | 0.703 |
| Excl. Hgb <9 g/dL | 6145 | 450 | 0.990 | 1.265 | 1.303 | 0.879 | 0.862 | 0.670 | 0.686 |
| Excl. MCHC <30 g/dL | 6485 | 482 | 0.989 | 1.269 | 1.304 | 0.883 | 0.873 | 0.676 | 0.669 |
| Excl. both | 6082 | 442 | 0.990 | 1.285 | 1.298 | 0.877 | 0.862 | 0.678 | 0.669 |
| Excl. mechanical ventilation | 6119 | 414 | 0.988 | 1.276 | 1.303 | 0.908 | 0.859 | 0.740 | 0.739 |
| Excl. unspecified stroke | 4576 | 340 | 0.988 | 1.175 | 1.266 | 0.825 | 0.894 | 0.698 | 0.705 |

Supplementary Table 6. Summary of sensitivity analyses

Notes: OR, odds ratio; Hgb, hemoglobin; MCHC, mean corpuscular hemoglobin concentration; GCS, Glasgow Coma Scale. All sensitivity analyses retained the same five predictors identified by LASSO regression. MCHC remained an independent predictor across all analyses (all *P*<0.05). Model discrimination remained moderate (AUC 0.67–0.74) in every sensitivity analysis, and all five predictors retained directionally consistent effects.
